# Genetics of pre-harvest sprouting resistance in a cross of Canadian adapted durum wheat genotypes

**DOI:** 10.1007/s11032-013-0006-y

**Published:** 2014-01-03

**Authors:** A. K. Singh, R. E. Knox, J. M. Clarke, F. R. Clarke, A. Singh, R. M. DePauw, R. D. Cuthbert

**Affiliations:** 1 Semiarid Prairie Agricultural Research Center, Agriculture and Agri-Food Canada, Swift Current, SK Canada; 2 Present Address: Department of Agronomy, Iowa State University, Ames, IA USA; 3 University of Saskatchewan, Saskatoon, SK Canada

**Keywords:** Wheat, Durum, Sprouting, QTL, Yield

## Abstract

Severe losses attributable to pre-harvest sprouting (PHS) have been reported in Canada in recent years. The genetics of PHS resistance have been more extensively studied in hexaploid wheat and generally not using combinations of elite agronomic parents. The objective of our research was to understand the genetic nature of PHS resistance in an elite durum cross. A doubled haploid (DH) population and checks were phenotyped in replicated trials for grain yield and PHS traits over 3 years in western Canada. The response of intact spikes to sprouting conditions, sampled over two development time points, was measured in a rain simulation chamber. The DH population was genotyped with simple sequence repeat and Diversity Arrays Technology markers. Genotypes were a significant source of variation for grain yield and PHS resistance traits in each tested environment. Transgressive segregant DH genotypes were identified for grain yield and PHS resistance measurements. Low or no correlation was detected between grain yield and PHS, while correlation between PHS resistance measurements was moderate. The heritability of PHS resistance was moderate and higher than grain yield. Significant quantitative trait loci with small effect were detected on chromosomes 1A, 1B, 5B, 7A and 7B. Both parents contributed to the PHS resistance. Promising DH genotypes with high and stable grain yield as well as PHS resistance were identified, suggesting that grain yield and PHS can be improved simultaneously in elite genetic materials, and that these DH genotypes will be useful parental material for durum breeding programs.

## Introduction

Pre-harvest sprouting (PHS) refers to germination of seeds in physiologically mature spikes prior to harvest. In Canada, PHS is reported to be a problem during wet or humid harvest conditions (McCaig and DePauw 1992). Severe losses were reported in three (2000, 2002 and 2010) of the past 10 years (Clarke et al. 2005a; Knox et al. 2012). The economic impact of PHS occurs through losses in grain yield, test weight, grain functionality and viability of seed for planting (Belderok 1968; Buchanan and Nicholas 1980; Czarnecki and Evans 1986; Derera 1989). Sprouted kernels are a grade determinant in Canadian durum wheat (*Triticum turgidum* subsp. *durum* (Desf.) Husn.). Samples with more than 0.5 % sprouted kernels are downgraded from a Canada Western Amber Durum (CWAD) No. 1 to CWAD No. 2, causing economic losses to producers, and more severe downgrading often occurs.

Sprouting in wheat produces the enzyme alpha-amylase, which leads to lower Hagberg falling number and influences cooked pasta quality. Sprouting also negatively affects vitreousness and number of damaged kernels (Dick et al. 1974). Grant et al. (1993) demonstrated that sprouting damage caused higher cooking losses, decreased firmness and lower spaghetti stickiness values. The texture of pasta made from grain affected by sprouting gets softer, and more starch is lost to cooking water, making the water cloudy. High levels of sprout damage can cause processing production problems such as uneven extrusion, strand stretching and irregular drying, leading to cracking of strands during storage (Donnelly 1980).

Pre-harvest sprouting resistance is a complex trait affected by environmental cues and is not easy to characterize (Gerjets et al. 2010; Knox et al. 2012). Most research concludes that the trait is quantitatively inherited and shows significant interaction with the environment. Genetic studies suggest that the trait is controlled by several genes or quantitative trait loci (QTL; for example, Ogbonnaya et al. 2008; Fofana et al. 2009; Rasul et al. 2009; Knox et al. 2012). The majority of the genetic studies on PHS have been conducted on hexaploid wheat and fewer in durum wheat, even though sprouting susceptibility is also an issue in durum. The level of PHS resistance in white seed coat hexaploid wheat and amber-seeded durum wheat cultivars was found to overlap with that of red-seeded wheat (McCaig and DePauw 1992). Several loci associated with PHS resistance in durum wheat have been reported previously in hexaploid wheat (Knox et al. 2012). Postulating durum genes based on hexaploid wheat genetics may be possible, but although durum and hexaploid wheat share the A and B genomes, the modern-day breeding for the two species has mainly been mutually exclusive. Numerous studies have reported the group 3 and 4 chromosomes as important carriers of genetic factors for PHS resistance. Group 3 QTL are not relevant because amber durum wheat does not possess the red seed coat genes which provide PHS resistance (Groos et al. 2002). Group 4 contains the *Phs* loci, which increases dormancy [reviewed in Flintham et al. (2002)] and may be relevant to durum. Good sources of PHS resistance have been identified, particularly in hexaploid wheat. However, often such sources of resistance are not easily transferred because of the unadapted nature of the genetic background. Variability does exist within adapted Canadian durum germplasm and the potential exists to recombine loci for enhanced sprouting resistance without major setbacks in breeding value. It is important to identify relevant and useful sources of PHS resistance from elite adapted genotypes of durum wheat to accelerate the breeding progress.

The two durum wheat genotypes DT696 and DT707 combine higher grain yield with other desirable traits and were tested in recent Canada Western Amber Durum registration trials. These genotypes are adapted to the drier prairie, and in preliminary studies showed promise for PHS resistance differences. A doubled haploid (DH) mapping population from these parents was therefore considered relevant to understanding the genetics of PHS resistance in elite parents, as well as providing an opportunity to make genetic gains for PHS resistance combined with agronomic performance and end-use quality breeding objectives. In contrast, the alternatives are to explore the use of unadapted or wild relatives of wheat with PHS resistance. Use of DH populations in mapping wheat offers several advantages including homogeneity within genotypes, immortal lineage for multiple tests and rapid development. Previously, DH mapping populations in durum wheat using the maize pollen method (Knox et al. 2000) have been used to map diverse traits: for example, grain pigment (Singh et al. 2009), grain Cadmium (Knox et al. 2009) and disease resistance (Singh et al. 2012a). A DH population should therefore be advantageous for the study of PHS resistance in durum. The objective of our research was to understand the genetic nature of PHS resistance across environments in an elite durum cross and the potential for simultaneous selection for PHS resistance and high stable grain yield.

## Materials and methods

### Genetic materials and field experiments

DT707 (PHS moderately susceptible) and DT696 (PHS resistant) were crossed and 122 DH genotypes were developed (Knox et al. 2000). DT707 derives from the cross AC Avonlea/DT665, and is a sib of the widely grown cultivar Strongfield (Clarke et al. 2005b). DT696 has the pedigree DT618/DT637//Kyle; Kyle (Townley-Smith et al. 1987) was widely grown previous to Strongfield. The population of DT707/DT696 was grown over 3 years (2005, 2006 and 2007) near Swift Current, Canada. The design each year was a two-replicate alpha-lattice with 17 blocks. Each experimental plot was four rows 0.9 m wide and 4 m long, which was trimmed to 3 m at maturity. Each plot was seeded with 800 seeds, with a seed drill at a depth of 5–6 cm. The experimental site was a Swinton loam (Orthic Brown Chernozem).

### Data collection

In each year and from each plot, 10 spikes were sampled when 50 % of the primary tillers in that plot had collapsed nodes on the stems, which was designated as sampling time 1 (T1; DePauw et al. 2009). Care was taken to ensure that spikes were representative of the plot from which they were taken and that the nodes were collapsed on the stem. Spikes showing prematurity blight or other disease problems were avoided, and an attempt was made to take spikes from primary culms. The spikes were hand-harvested by cutting the peduncle about 12 cm below the base of the spike. Labels were used to tie and keep the spikes together, then they were placed in labeled boxes and stored in a freezer at −23 °C immediately after collection until threshing to minimize metabolic activity that would cause a loss of dormancy by after-ripening (Noll and Czarnecki 1980). In each year, a second set of spikes was collected 10 days after the first set (T2) and stored as described above. At maturity, each plot was harvested using a small plot combine and grain yield (kg ha^−1^) was recorded.

### Rain simulator

The response to sprouting conditions of intact spikes was measured by providing a uniform wetting treatment in a rain simulation chamber (DePauw and McCaig 1991). Each bundle of 10 spikes was placed upright on a tray fitted with wire mesh on a 0.5-cm grid. After an initial wetting treatment of about 135 mm in 5 h, water was sprayed for 0.5 h every 12 h for 5 days, and at that time the spikes were removed for scoring. Ambient temperature was maintained at 18 °C and relative humidity greater than 95 %. The number of spikes per bundle with visible evidence of germination was recorded [according to Knox et al. (2012)] for the bundles collected at T1 (T1HS) and at T2 (T2HS). Percent kernels sprouted in the T1 bundle (T1KS) and the T2 bundle (T2KS) were measured based on the threshed kernels.

### Molecular marker evaluation

The DH population was genotyped with 122 simple sequence repeat (SSR) markers which were polymorphic on the parents. Several hundred SSR were used to identify polymorphism between the parental genotypes. The population was further characterized with 112 polymorphic Diversity Arrays Technology (DArT) markers picked after a full DArT array screening. The DNA was extracted from parents and DH genotypes for PCR using the Wheat and Barley DNA Extraction in 96-well Plates protocol (http://maswheat.ucdavis.edu/PDF/DNA0003.pdf) with modifications. When the plants reached the 1–2 leaf stage, 3-cm leaf segments from primary leaves were harvested for genomic DNA isolation. A 10-μl PCR reaction consisting of DNA (final concentration of 20 ng/μl), Ultrapure Distilled H_2_O (Gibco), 10 %—10× PCR buffer without MgCl_2_ [Invitrogen cat.#18067-017: 200 mM Tris–HCl (pH 8.4), 500 mM KCl], 10 mM dNTPs (Roche), 1.5 mM MgCl_2_ (Invitrogen), 0.07 U/μl *Taq* (5 U of activity/μl) NEB, and 2 ng/μl forward and 2 ng/μl reverse primer was used for the DNA amplification process. PCR conditions were an initial denaturation at 94 °C for 3 min, followed by 44 cycles of 94 °C for 1 min, 55 or 60 °C annealing for 1 min and 72 °C extension for 1 min, with a final extension at 72 °C for 10 min. The amplification products were resolved by electrophoresis using an ABI3730xl DNA fragment analyser (Applied Biosystems), or mixed 2 % Metaphor and 1 % agarose LE gels run at 4 V cm^−1^ in TBE (0.045 M TRIS, 0.045 M borate, and 0.001 M EDTA) buffer and stained with ethidium bromide (0.5 μg/ml). The size of bands was determined by comparing against a 50-bp DNA ladder. The DNA banding patterns were visualized with UV light and recorded by a Kodak Gel Logic 100 digital camera imaging system.

DArT genotyping was done by Triticarte Pvt. Ltd. (Yarralumla, ACT, Australia; www.triticarte.com.au). DNA was extracted from parents and DH genotypes for DArT analysis according to the protocol published by Triticarte. Briefly, a genomic representation of a mixture of the entire population was produced with *Pst*I–*Taq*I digestion, spotted on microarray slides, and the individual genotypes were evaluated for polymorphism based on fluorescence signals.

### Statistical analyses

Analysis of variance (ANOVA) was done on each environment separately in SAS v9. Genotypes were considered fixed effects, while blocks and replicates were considered random effects. Assumptions of the ANOVA were tested through the PROC Univariate Shapiro–Wilk statistic for normality of residuals and through a plot of predicted*residuals for homogeneity of residuals. Outliers were tested using the Studentized residuals (Lund 1975). A type I error rate of 0.05 was used for all analysis. Least square means for each genotype for traits measured was used for QTL analysis.

The Lin and Binn superiority index measure was used to identify the best performing genotype in tested environments; it is an approach to measuring superiority that combines performance and stability (Lin and Binns 1988). The superiority measure was calculated as:

$$ P_{i} = \sum\limits_{j = 1}^{n} {\left( {X_{ij} {-}M_{j} } \right)^{2} /2n} $$

where *P*_*i*_ = superiority index of the *i*th cultivar, *X*_*ij*_ = response variable of the *i*th cultivar in the *j*th environment, *M*_*j*_ = maximum response obtained among all the cultivars in the *j*th environment and *n* = number of environments. Response variables included grain yield, T1HS, T2HS, T1KS and T2KS.

The lower *P*_*i*_ value was considered as superior using the P1 cutoff. The cutoff point for *P*_*i*_ values was ${\text{MS}}_{\text{residual}} \times F_{{{\text{df}}(g),(g{-}1)(e{-}2)}} .$

Broad-sense heritability and confidence intervals were calculated for grain yield, T1HS, T2HS, T1KS and T2KS according to Knapp et al. (1985).

### QTL mapping

A genetic linkage map was constructed using the software JoinMap 4.0 using the regression mapping option and groupings were created using independence LOD (Van Ooijen 2006). Centimorgan (cM) values were calculated according to the Kosambi mapping function. Each linkage group was assigned to the corresponding durum wheat chromosome based on the known genomic positions of the DArT and SSR markers in the groups. This was accomplished by utilizing integrated maps (Mantovani et al. 2008; Marone et al. 2012) and the GrainGenes website. QTL mapping was performed using MapQTL.6 (Van Ooijen 2009) to identify molecular markers significantly associated with QTL for grain yield, T1HS, T2HS, T1KS and T2KS. Logarithm of the odds (LOD) threshold for significance was obtained by MapQTL’s permutation test option (1,000 permutations). Genome-wide threshold levels were used to declare significant QTL based at a 5 % significance level. Automatic co-factor detection based on backward elimination as well as manual co-factor selection was used to identify the co-factor markers for multiple QTL mapping (MQM).

## Results

Genotypes were a significant source of variation for grain yield, T1HS, T2HS, T1KS and T2KS in each tested environment (Table 1). While the grain yield of the parents, DT696 and DT707, was not significantly different in the tested environments, the DH progenies showed transgressive segregation. Transgressive segregant with higher grain yield than the parents were noted in two (2006 and 2007) out of 3 years. The parents differed in level of PHS resistance and depending on the measure a significant difference was observed in each of the 3 years. For example, the parents were significantly different in 2005 and 2006 for T1HS, and in 2006 and 2007 for T2HS. Considering both sampling times, in years when the parents were not significantly different for Heads Sprouted they were significantly different for Kernels Sprouted. DH genotypes showed transgressive segregation with significantly lower sprouting values than the lowest parent in each year, depending on the measure. Depending on year, significant transgressive segregation for PHS resistance was observed for both T1 and T2. Significant moderately positive correlations were found between all four PHS measurements: T1HS, T2HS, T1KS and T2KS (Table 2). While no significant correlation was present between grain yield and the four PHS traits in 2005, low significant correlations were noted for 2006 and 2007. Heritability of grain yield ranged from 0.41 to 0.64, T1HS ranged from 0.55 to 0.70, T2HS ranged from 0.57 to 0.65, T1KS ranged from 0.50 to 0.55 and T2KS ranged from 0.54 to 0.75.

**Table 1:** Performance of the DT707/DT696 durum DH population and parents grown in 2005, 2006 and 2007 near Swift Current, Canada, for grain yield, T1 Heads Sprouted (T1HS), T2HS, T1 Kernels Sprouted (T1KS) and T2KS

	Year	LS means range (population)	Mean (DT696)	Mean (DT707)	LSD_(0.05)_	Parents significantly different	Significantly transgressive segregant from	
							Low parent	High parent
Grain yield (kg/ha)	2005	2,764–3,733	3,646	3,350	354	No	Yes	No
	2006	2,481–3,335	2,928	2,932	332	No	Yes	Yes
	2007	1,471–2,251	1,736	1,973	238	No	Yes	Yes
T1HS (heads 0–10)	2005	1.5–10.0	2.5	8.5	2.9	Yes	No	No
	2006	0.1–9.4	1.0	7.3	3.9	Yes	No	No
	2007	1.4–10.0	8.1	10.3	3.5	No	Yes	No
T2HS (heads 0–10)	2005	1.5–10.0	6.0	8.0	3.3	No	Yes	No
	2006	0.0–9.5	2.5	7.0	3.5	Yes	No	No
	2007	1.0–10.0	4.0	9.4	3.9	Yes	No	No
T1KS (%)	2005	17.0–60.0	26.0	43.0	20.6	No	No	No
	2006	1.0–47.0	9.0	20.0	17.5	No	No	Yes
	2007	6.6–65.6	38.2	65.5	22.9	Yes	Yes	No
T2KS (%)	2005	17.9–62.7	39.4	61.2	19.0	Yes	Yes	No
	2006	0.0–51.7	19.0	17.8	13.1	No	Yes	Yes
	2007	5.2–53.9	32.2	18.4	20.0	No	No	Yes

**Table 2:** Pearson’s correlation between grain yield, T1 Heads Sprouted (T1HS), T2HS, T1 Kernels Sprouted (T1KS) and T2KS from the DT707/DT696 durum DH population grown in 2005, 2006 and 2007 near Swift Current, Canada

Year	Trait	T1HS	T2HS	T1KS	T2KS
2005	Yield	ns	ns	ns	ns
2006		0.26**	0.19*	0.21*	0.18*
2007		0.18*	0.24**	0.22**	0.17*
2005	T1HS		0.58***	0.62***	0.46***
2006			0.63***	0.67***	0.56***
2007			0.60***	0.76***	0.61***
2005	T2HS			0.59***	0.64***
2006				0.64***	0.75***
2007				0.58***	0.70***
2005	T1KS				0.62***
2006					0.72***
2007					0.73***

*0.05; **0.01; ***0.001

The Lin and Binns superiority index (*P*_*i*_), as a combined measure of stability and performance, identified a few DH genotypes from the mapping population with superior PHS resistance that were the same as the highest grain yield genotypes. These DH genotypes were not only high-yielding relative to the population, their performance was consistent across the three environments. For example, the DH genotype A0132&AA047 ranked 24th for yield in 2005, 30th in 2006 and second in 2007 out of the 122 genotypes in the population. A0132&AA047 was of particular interest not only for its superior performance for grain yield but also for pre-harvest sprouting resistance traits T2HS, T1KS and T2KS (Table 3). A number of other genotypes were identified for their superior PHS resistance based on the superiority index. Four DH genotypes, A0132&AM052, A0132&BD013, A0132&BQ079 and A0132&CC058, were superior for T1HS, T2HS, T1KS and T2KS. However, in this group of four genotypes, A0132&CC058 would be less desirable than the other three for further breeding purposes due to lower grain yield (Table 3).

**Table 3:** Performance of select genotypes from the DT707/DT696 DH population evaluated in 2005, 2006 and 2007 near Swift Current, Canada, compared to the best performing genotypes for grain yield and pre-harvest sprouting traits

Genotype	Grain yield (kg/ha)	Superiority index (*P*_*i*_)				
		Grain yield	T1HS	T2HS	T1KS	T2KS
A0132&AA047	2,882	*		*	*	*
A0132&BL024	2,825	*		*		
A0132&BD067	2,769			*	*	*
A0132&AM052	2,742		*	*	*	*
A0132&AF031	2,708			*	*	*
A0132&BQ079	2,705		*	*	*	*
A0132&CC045	2,671		*	*		*
A0132&BD013	2,621		*	*	*	*
A0132&AB036	2,508			*	*	*
A0132&BB013	2,489			*	*	*
A0132&CC058	2,441		*	*	*	*
DT696	2,755					
DT707	2,748					

Comparisons were made using the Lin and Binns superiority index (*P*_*i*_)

* Genotype was not significantly different from the best performing genotypes in the population based on superiority index

**Fig. 1:**
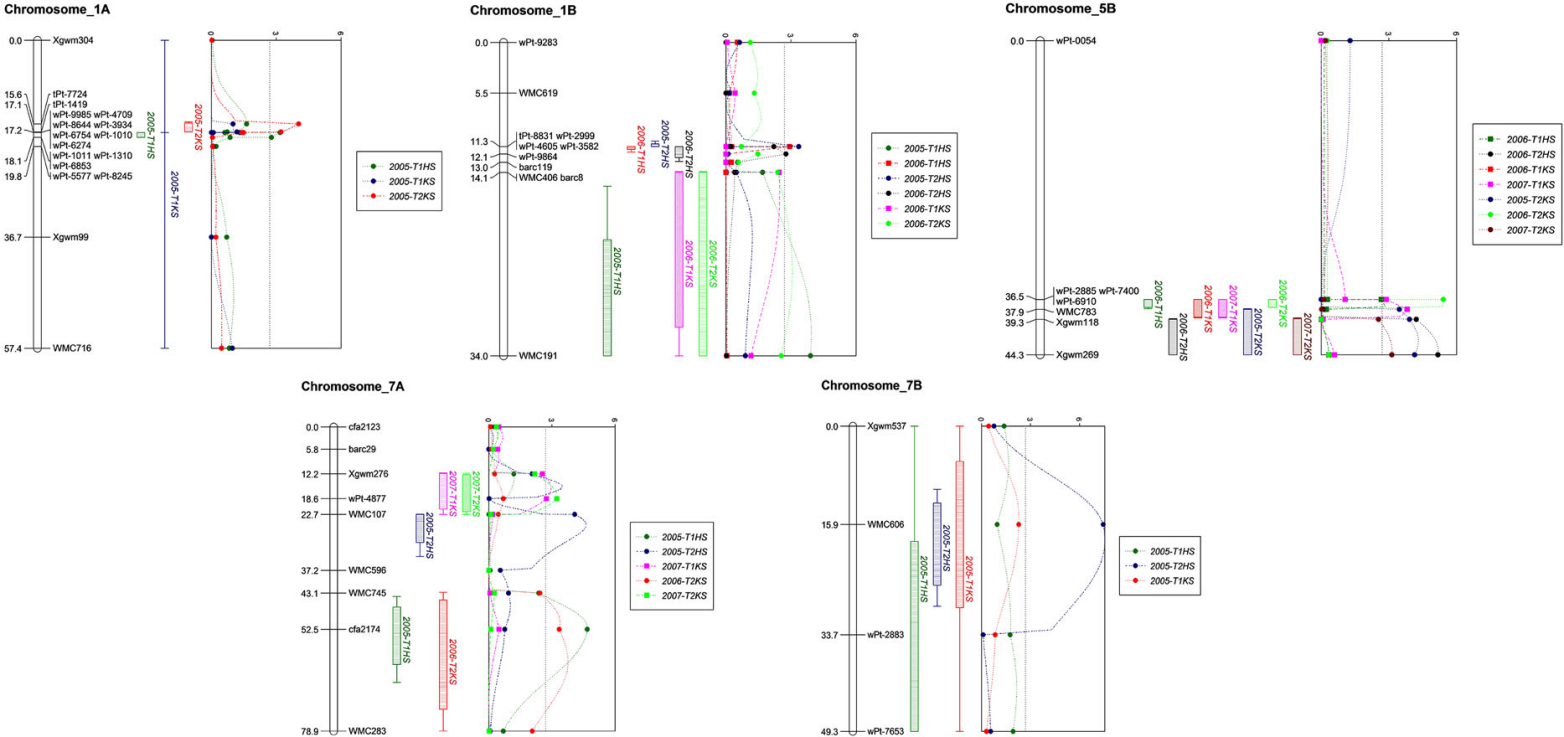
Significant pre-harvest sprouting QTL identified from an elite cross of durum wheat (DT707/DT696) using a DH mapping population grown near Swift Current, Canada, in 2005, 2006 and 2007 in replicated tests. PHS QTL were identified over two development-stage time points (T1 and T2) and for Heads Sprouted (HS) and Kernels Sprouted (KS)

Twenty-four linkage groups were constructed that contained at least three markers. Groups with two markers were considered as unmapped. The total coverage of the 24 linkage groups was 806 cM. In the mapping population, PHS resistance QTL were found on chromosomes 1A (*QPhs.spa*-*1A*), 1B (*QPhs.spa*-*1B*), 5B (*QPhs.spa*-*5B*), 7A (*QPhs.spa*-*7A*) and 7B (*QPhs.spa*-*7B*; Fig. 1). Both DT696 and DT707 contributed favorable alleles for lower PHS values (T1HS, T2HS, T1KS and T2KS; Table 4). DT696 contributed the PHS resistance allele for QTL on 1A, 1B and 7A, and DT707 contributed favorable QTL on 5B and 7B. The favorable allele consistently derived from the same parent across the four PHS trait measurements and environments for each QTL (Table 4). Multiple QTL mapping (MQM) analysis provided the most likely marker position of the QTL (Table 4). Most of the PHS resistance QTL produced a small effect with less than 15 % of phenotypic variation (*R* ^2^) explained, which was consistent with the moderate heritabilities. Effects of the 5B and 7A PHS resistance QTL were observed in all years, whereas QTL on 1B were observed in 2 years. The 1A and 7B QTL were observed in only 1 year. Due to the superior grain yield, stability and PHS resistance of A0132&AA047, we dissected the molecular variant haplotype for all significant chromosomes for this genotype and determined that in all cases this genotype carries the favorable parental molecular variant of the significant markers.

**Table 4:** Chromosomal location, peak marker position, LOD, phenotypic variation, additive effect and parental source of favorable allele of significant QTL from the DT707/DT696 durum wheat DH population identified by multiple QTL mapping (MQM) using MapQTL for T1 heads sprouted (T1HS), T2 heads sprouted (T2HS), T1 kernels sprouted (T1KS) and T2 kernels sprouted (T2KS) from experiments grown in 2005, 2006 and 2007 near Swift Current, Canada, in replicated tests

Environment/measure	Chromosome	Marker with most likely position	LOD	Phenotypic variation *R* ^2^ (%)	Additive effect	Parental source of favorable allele	QTL designation
2005/T1HS	1A	*wPt*-*6274*	3.2	8.7	–0.58	DT696	*QPhs.spa*-*1A*
2005/T1HS	1A	*wPt*-*1011*	2.8	7.7	–0.54	DT696	*QPhs.spa*-*1A*
2005/T2KS	1A	*tPt*-*7724*	4.0	11.2	–3.46	DT696	*QPhs.spa*-*1A*
2005/T2KS	1A	*tPt*-*1419*	3.2	9.0	–3.10	DT696	*QPhs.spa*-*1A*
2005/T1HS	1B	*Xwmc191*	3.9	11.0	–0.69	DT696	*QPhs.spa*-*1B*
2005/T2HS	1B	*tPt*-*8831*	3.4	8.4	–1.21	DT696	*QPhs.spa*-*1B*
2006/T1HS	1B	*wPt*-*4605, wPt*-*3582*	2.9	8.6	–0.63	DT696	*QPhs.spa*-*1B*
2006/T2HS	1B	*wPt*-*9864*	2.8 ns	8.2	–0.55	DT696	
2006/T2KS	1B	*wPt*-*9283*	3.5	9.0	–2.47	DT696	*QPhs.spa*-*1B*
2006/T1KS	1B	*Xwmc406, Xbarc8*	2.5 ns	7.8	–2.38	DT696	
2006/T1HS	5B	*wPt*-*6910, wPt*-*7400*	2.7 ns	7.8	0.60	DT707	
2005/T2KS	5B	*Xwmc783*	3.5	9.9	3.25	DT707	*QPhs.spa*-*5B*
2005/T2KS	5B	*Xgwm118*	3.9	11.1	3.45	DT707	*QPhs.spa*-*5B*
2005/T2KS	5B	*Xgwm269*	4.1	11.7	3.57	DT707	*QPhs.spa*-*5B*
2006/T1KS	5B	*Xwmc783*	3.8	12.4	3.02	DT707	*QPhs.spa*-*5B*
2006/T1KS	5B	*wPt*-*6910*	2.9	9.5	2.64	DT707	*QPhs.spa*-*5B*
2006/T2HS	5B	*Xgwm118*	4.2	13.3	0.71	DT707	*QPhs.spa*-*5B*
2006/T2HS	5B	*Xgwm269*	5.2	16.1	0.78	DT707	*QPhs.spa*-*5B*
2006/T2KS	5B	*wPt*-*6910, wPt*-*7400*	5.4	14.4	3.12	DT707	*QPhs.spa*-*5B*
2007/T1KS	5B	*Xgwm118*	2.7	7.6	3.61	DT707	*QPhs.spa*-*5B*
2007/T1KS	5B	*Xgwm269*	4.0	10.9	4.36	DT707	*QPhs.spa*-*5B*
2007/T2KS	5B	*Xgwm269*	3.1	9.6	3.32	DT707	*QPhs.spa*-*5B*
2005/T1HS	7A	*Xcfa2174*	4.7	13.4	–0.84	DT696	*QPhs.spa*-*7A*
2005/T2HS	7A	*Xwmc107*	4.1	10.4	–1.51	DT696	*QPhs.spa*-*7A*
2006/T2KS	7A	*Xcfa2174*	3.3	8.6	–2.55	DT696	*QPhs.spa*-*7A*
2007/T1HS	7A	*Xcfa2174*	2 ns	7.4	–0.57	DT696	
2007/T2KS	7A	*wPt*-*4877*	3.2	9.9	–3.32	DT696	*QPhs.spa*-*7A*
2007/T1KS	7A	*wPt*-*4877*	2.7	7.3	–3.51	DT696	*QPhs.spa*-*7A*
2005/T2HS	7B	*Xwmc606*	7.5	20.2	0.95	DT707	*QPhs.spa*-*7B*

## Discussion

The results of transgressive segregation, moderate heritability and correlations among measures, differences in PHS resistance between sampling times and years, and multiple QTL provide evidence that PHS resistance is under complex genetic control within the DT707/DT696 population. The observation of transgressive segregants for greater PHS resistance suggests a genetic contribution from both parents. Due to the complex genetic control and influence of environment on trait expression, there is a need to simultaneously consider different measures and to make measurements at different stages and in multiple environments. Knox et al. (2012) had previously concluded the necessity of considering multiple types of measurements at different intervals of sprouting in order to get a more complete understanding of the genetic factors controlling PHS resistance. In other words, one form of measurement will not sufficiently characterize this complex trait. Despite the apparent complexity of the PHS resistance trait, our results also indicated that it was possible to recombine and recover desirable transgressive segregants.

The Heads Sprouted measure (T1HS and T2HS) focuses on sprouting resistance that includes head characteristics, whereas the Kernels Sprouted (T1KS and T2KS) measure focuses on dormancy. One could infer that different facets of PHS resistance are being characterized based on the inconsistency in response observed across environments and sampling times between HS and KS in Table 1. However, difference in precision of the two measurements could also be an explanation for the inconsistencies. For example, no transgressive segregation was identified in 2006 for HS, whereas transgressive segregation occurred for KS in that year. There is ample evidence of complex genetic interactions with environment for PHS (see, for example, Gerjets et al. 2010; Knox et al. 2012). The early (T1HS and T1KS) and late (T2HS and T2KS) sampling times provide insight into the durability of sprouting resistance (DePauw et al. 2012), and a measure of the effect of longer exposure of spikes to the field environment. The use of two time points (T1 and T2) provides a mechanism to test the effectiveness of genetic factors governing PHS resistance, such as length of the dormancy period, on their usefulness against sustained environmental weathering. Phenological differences play a major role in confounding PHS resistance with heading or maturity. By stratifying our sample collection on a plant developmental growth stage (collapsed node), we minimized the influence of phenological differences. Collapsed nodes of wheat stems are associated with about 16 % grain moisture (Knox et al. 2012), which allows the grain to be stored and provides a level of after-ripening that allows a differential response in dormancy to be observed. While we minimized the phenological differences by sampling at collapsed node stage, it will be desirable to generate further information on days to maturity QTL and determine if these are coincident with PHS resistance QTL.

Moderate correlations among PHS measures are expected (Knox et al. 2005). The KS samples came from HS spikes, and therefore seed dormancy is a factor in both measures. The moderate correlation between T1 and T2 indicates that there may be genetic factors that control PHS resistance through periods of prolonged exposure to weathering conditions. The lack of high correlation among measures is another indication that different genetic factors are involved between the two phenotypes of HS and KS. The moderate correlation between T1 and T2 can be explained by after-ripening, whereby dormancy declines over time. The significance of QTL on multiple chromosomes for T1 and T2 suggested that these QTL are important in extended sprouting resistance throughout a protracted harvest period where there is greater potential to undergo a weathering event. The expression of the QTL, such as *QPhs.spa*-*5B* and *QPhs.spa*-*7A*, in multiple environments and across sampling times T1 and T2 suggested an opportunity to bring them together to provide a useful level of PHS resistance.

The 1A QTL we identified is in a similar region to that reported by Singh et al. (2010), where they hypothesized that their QTL could be associated with alpha-amylase activity and not with grain dormancy per se. Two independent PHS resistance QTL have been identified on this chromosome (Anderson et al. 1993). Pre-harvest sprouting resistance QTL on 1A were previously reported by Munkvold et al. (2009), and these are in a similar genomic region to that reported in Canadian genetic material (Knox et al. 2012; Semagn et al. 2006). Gelin et al. (2006) also reported a PHS resistance QTL on 1A, but distal to the QTL identified by Knox et al. (2012).

Pre-harvest sprouting resistance QTL on 1B were reported using association mapping (Kulwal et al. 2012) and linkage mapping (Munkvold et al. 2009). This is likely the same QTL as reported previously (Knox et al. 2012; GrainGenes website). It is worth noting that in these two studies, as well as in our study, the PHS resistance QTL do not have a large effect.

QTL for PHS resistance were reported in Canadian hexaploid wheat on chromosome 5B (Fofana et al. 2008). The PHS-resistant genotype RL4137 had a significant QTL around *Xwmc537*, which was suggested to be informative for alleles associated with PHS resistance QTL on 5B. Pre-harvest sprouting resistance QTL in Australian wheat were also identified on 5B. Four of five markers which we identified in the linkage group were previously reported in GrainGenes (http://wheat.pw.usda.gov) or Triticarte as mapping to 5B, and also by Ghavami et al. (2011). However, marker *Xgwm269* is reported to be on 4A and 5D according to the GrainGenes website. Our grouping showed a 4-cM distance between *Xgwm269* and its closest marker on 5B, and the latter marker did not group with the other 4A markers. However, it cannot be concluded that *Xgwm269* is definitely on 5B. In a recent meta-QTL analysis study, on chromosome 4A *Xgwm269* was reported linked to PHS resistance (Tyagi and Gupta 2012). Fusarium head blight QTL mapped to DArT markers *wPt*-*2885*, *wPt*-*6910* and *wPt*-*7400* in durum wheat (Ghavami et al. 2011), the same region as *QPhs.spa*-*5B*. It is worth pursuing further study of this genomic region to determine whether PHS and FHB QTL can be bred together.

The PHS resistance QTL on 7A has been reported in white grain hexaploid wheat close to *Xbarc222*, which is closely linked to *Xcfa2174* (Singh et al. 2010). In our study, we found two QTL on 7A; however, further genetic studies using bigger populations and more environments will be required to confirm whether there are two separate QTL on 7A. The QTL on 7A, with a peak LOD at *Xwmc107*, is near *Xgwm276*, which was identified by Knox et al. (2012) as a site for PHS resistance. Based on the Kofa × UC1113 map on GrainGenes, the PHS resistance QTL on 7B at marker *Xwmc606* is linked to *Xbarc72*, which was identified by Knox et al. (2012) as being linked to PHS resistance.

The QTL on chromosomes 1A, 1B, 5B and 7A were identified for several trait-measures by environment combinations. The QTL on 7B, although notably with the highest LOD, only occurred in one trait-measure by environment combination. While we did not study the underlying biology of PHS resistance expression, it is important to note that a previous study reported ABA responsiveness QTL in genomic regions overlapping with our 1A and 7B PHS QTL (Kobayashi et al. 2010). These results, along with previously reported co-localization of PHS QTL, suggest most of these QTL have an important role in PHS resistance in the tested environment and that enriching breeding populations for these QTL using a marker-assisted approach would be worthwhile. On the other hand, no single marker was significant for all trait-measure by environment combinations, providing evidence of the complexity of the trait and the need to deploy multiple loci.

The two parents, DT707 and DT696, share a portion of their pedigree, and therefore they will likely have identity-by-descent. Additionally, they likely share identity-by-state. Higher IBD and IBS were demonstrated by lack of marker polymorphism between the two parents. Such parents have advantages and disadvantages when used to develop a mapping population. With shared pedigree, the level of polymorphism is low; therefore dense marker coverage is unlikely and this is what we experienced with the DT707/DT696 population. On the other hand, identity-by-descent will reduce the background noise from many minor gene differences (Singh et al. 2011). Although the two parents, DT707 and DT696, did not differ for grain yield in the 3 years of testing, the observed transgressive segregation in 2 years indicated genetic differences for grain yield. The low but positive correlation observed between grain yield and PHS resistance measures is useful in plant breeding as it indicates that these two traits are independent and can be improved simultaneously.

By evaluating the DH population in replicated testing in multiple environments, we were able to develop an understanding of the grain yield performance and stability along with the measurement of PHS. The information generated is useful for selecting the best yielding stable genotype with the desirable PHS resistance. Using the superiority index, the genotype A0132&AA047 was identified to be the best performing among the entire groups of DH genotypes from the DT707/DT696 population. This genotype is a good candidate for simultaneous improvement of grain yield and PHS resistance, as it possesses the favorable molecular variant of all PHS resistance QTL identified in our study along with a high and stable grain yield. A number of other genotypes were also identified with good performance in all four PHS resistance parameters, demonstrating their usefulness as parents in improving PHS tolerance in durum wheat. One of the DH genotypes from this population, Transcend, was registered for commercial production and in its pre-commercialization testing was noted as an improvement over the current predominant cultivars for Hagberg falling number (Singh et al. 2012b), one of the indirect assessments of sprouting (DePauw et al. 2012). Obviously, to introduce new variation, wider crosses will be required, but our study confirms that the potential exists to improve PHS resistance by intercrossing adapted durum genotypes.
